# A Chemically Patterned Microfluidic Paper-based Analytical Device (C-µPAD) for Point-of-Care Diagnostics

**DOI:** 10.1038/s41598-017-01343-w

**Published:** 2017-04-26

**Authors:** Trinh Lam, Jasmine P. Devadhasan, Ryan Howse, Jungkyu Kim

**Affiliations:** 1 Department of Chemical Engineering, Texas Tech University, Lubbock, TX USA; 2 Department of Mechanical Engineering, Texas Tech University, Lubbock, TX USA

## Abstract

A chemically patterned microfluidic paper-based analytical device (C-µPAD) is developed to create fluidic networks by forming hydrophobic barriers using chemical vapor deposition (CVD) of trichlorosilane (TCS) on a chromatography paper. By controlling temperature, pattern size, and CVD duration, optimal conditions were determined by characterizing hydrophobicity, spreading patterns, and flow behavior on various sized fluidic patterns. With these optimal conditions, we demonstrated glucose assay, immunoassay, and heavy metal detection on well-spot C-µPAD and lateral flow C-µPAD. For these assays, standard curves showing correlation between target concentration and gray intensity were obtained to determine a limit of detection (LOD) of each assay. For the glucose assays on both well-spot C-µPAD and lateral flow C-µPAD, we achieved LOD of 13 mg/dL, which is equivalent to that of a commercial glucose sensor. Similar results were obtained from tumor necrosis factor alpha (TNFα) detection with 3 ng/mL of LOD. For Ni detection, a colorimetric agent was immobilized to obtain a stationary and uniform reaction by using thermal condensation coupling method. During the immobilization, we successfully functionalized amine for coupling the colorimetric agent on the C-µPAD and detected as low as 150 μg/L of Ni. These C-µPADs enable simple, rapid, and cost-effective bioassays and environmental monitoring, which provide practically relevant LODs with high expandability and adaptability.

## Introduction

A microfluidic paper-based analytical device (µPAD) presents a promising alternative to traditional laboratory tests by allowing for the rapid and low-cost diagnoses of diseases in resource-limited settings. Recently, µPADs have received considerable attention since the world health organization (WHO) suggested that µPADs are promising diagnostic tools for the developing world^1–3^. µPADs offer many advantages as simple and portable platforms that require only a drop of target sample for detecting various analytes such as proteins, environmental contaminants, pathogens, chemicals, heavy metals, and drugs^4–9^. Unlike the active microfluidic devices, µPADs can transport target samples and reagents via capillary action without the need for mechanical components, external pumps, or controllers.

µPADs are fabricated by forming hydrophobic barriers onto various paper platforms^10^. Currently, several techniques have been reported to fabricate µPADs such as photolithography^5, 6, 11^, plotting with an analogue plotter^12^, ink jet etching^13, 14^, plasma treatment^15, 16^, paper cutting^17, 18^, wax printing^19–21^, inkjet printing^16, 22, 23^, flexography printing^24^, screen printing^25^, and laser patterning techniques^26^. The fundamental principle of these µPAD fabrication techniques is to form hydrophilic-hydrophobic barriers on a chromatography or filter paper to create fluidic channel networks. Liquid follows hydrophilic wicking matrices by capillary forces which can be predicted and analyzed by Lucas–Washburn and Darcy equations^27, 28^.

Although several µPAD fabrication techniques have been reported, each technique has its own drawbacks. For example, µPAD fabrication via wax printing is a simple, rapid, and cost effective method. However, this wax printed µPAD is unstable under high temperature and sensitive to organic solvents which can penetrate through wax barriers. Photolithography technique requires an extra washing step to remove un-crosslinked polymers^11^. Inkjet etching requires the paper substrate to be coated with polystyrene for 2 hours prior to printing to have high stability^13, 14^. Other µPAD fabrication techniques such as flexography printing and screen printing are sensitive to organic solvents as well^22^. In contrast, chemical modification techniques such as inkjet printing using alkyl ketene dimer (AKD) and plasma treatment result in proper solvent resistance since chemical agents effectively couple with hydroxyl groups on cellulose fiber in chromatography paper covalently^27^. However, the plasma treatment requires different masks for creating different microfluidic patterns on a paper and inkjet printing technique requires an extra heating step for eight minutes after AKD deposition^16, 22, 23^. Kwong *et al*. developed an initiated chemical vapor deposition (iCVD) method to deposit poly (methacrylic acid-co-ethylene glycol dimethacrylate) and poly (methacrylic acid) (PMMA) on a chromatography paper. However, the fabrication process requires more than 15 minutes including multiple washing steps to remove the ungrafted PMMA from the paper substrate^29^.

To resolve these drawbacks, we developed a chemical vapor deposition (CVD) method to create a thermally and chemically stable hydrophobic barrier for µPADs which is called “C-µPAD”. This C-µPAD fabrication technique requires a chromatography paper, trichlorosilane (TCS) as a hydrophobic agent, and a vacuum chamber to create hydrophobic patterns. The fabrication requires a single step that takes only two minutes to create a C-μPAD. Various C-µPADs were fabricated to show the versatility of this device by demonstrating glucose assay, immunoassay and heavy metal detection.

## Results

### Characterization of C-µPAD

**Figure 1:**
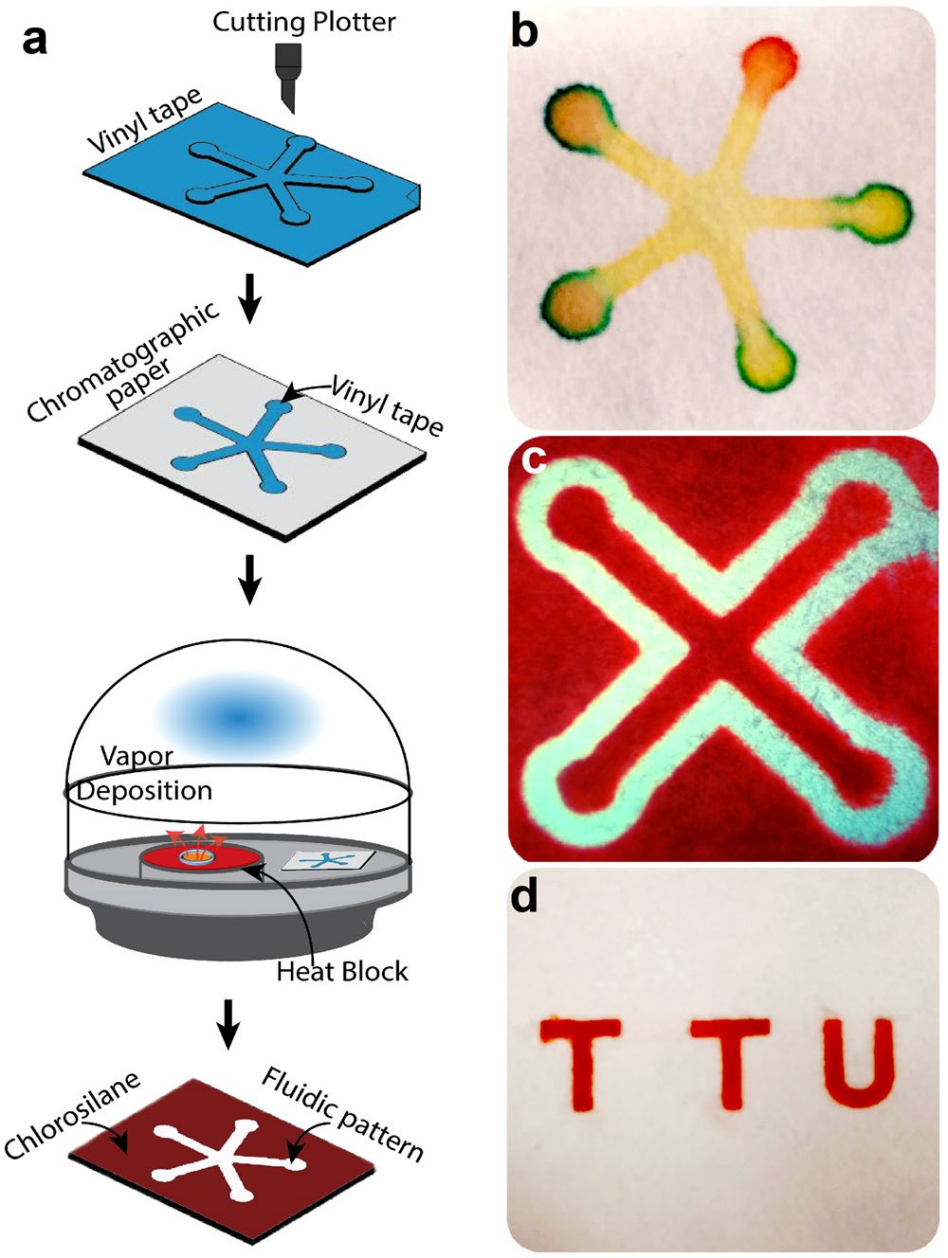
Development of paper-based microfluidic platform using C-µPAD technique. (**a**) Schematic illustration of fabrication process: A vinyl tape was cut based on a designed CAD file and then transferred onto 4.5 × 5 cm chromatography paper. The patterned paper was placed into the vacuum chamber with 100 µL of TCS solution placed on a 53 °C heat block. After vacuum process, the tape was removed and fluidic pattern was ready for bioassay. (**b**) and (**c**) Positive and negative features of 2 dimensional channels on C-µPAD (**d**) Demonstration of a multi-layered C-µPAD including top layer (TTU letters) and bottom layer (interconnection channels between letters).

In the C-µPAD fabrication, hydrophobic barriers were achieved by CVD process of TCS as shown in Fig. 1(a) to silanize a chromatography paper; the reaction took place readily with TCS vapor and required a low-pressure chamber and heat block as a source of heat supply^30^. In this study, vaporized TCS molecules from CVD process penetrated through the paper to form covalent bonds with hydroxyl groups on cellulose fibers that provided extremely stable and highly reproducible hydrophobic barriers. TCS is a highly volatile compound which has 594 mmHg of vapor pressure at 25 °C. The deposition of TCS molecules is strongly dependent on pressure, CVD duration, temperature, volume of TCS, and the mobility of the molecules. By controlling the duration and temperature in the CVD process, the chemical molecules will travel through the chromatography paper and immobilize uniformly throughout the paper. Thus, different CVD duration and temperature were followed to find the optimum. After CVD processes with different durations, contact angle measurement was used to demonstrate the strength of hydrophobic barrier of TCS. Previous studies demonstrated that the contact angle of the water droplet on a highly hydrophobic filter paper was 110~125°^31, 32^. In the C-µPAD fabrication process, the relationship of contact angle and the CVD duration was proportional until reaching the saturation point. The hydrophobicity of the C-µPAD surface was observed by an increase in the contact angle that is shown in Supplementary Figure S1. From 0 to 10 minutes of the CVD duration, the contact angle was 115° at 30 seconds and reached the saturation point of approximately 125° after 10 minutes. Moreover, the contact angle measurement ensured the quality and reproducibility of various C-µPADs. Hence, well-controlled hydrophilic barrier could be created by maintaining a standard contact angle. Based on our results, the contact angle of 115° on the top side of the pattern was stable and strong enough to carry out the bioassay. After the C-µPAD fabrication process, silanized hydrophobic patterns are invisible to the eyes and the modified area retains its original flexibility. Figure 1(b–d) shows various C-µPAD patterns on chromatography paper with food dyes. The patterns show both single layered C-µPAD with multiple color depositions and double layered C-µPAD. These C-µPADs exhibited the well-defined hydrophobic borders to wick uniformly throughout the hydrophilic paper networks. In addition, the aforementioned CVD process can be extended to fabricate the C-µPAD on other types of paper without any significant modification.

**Figure 2:**
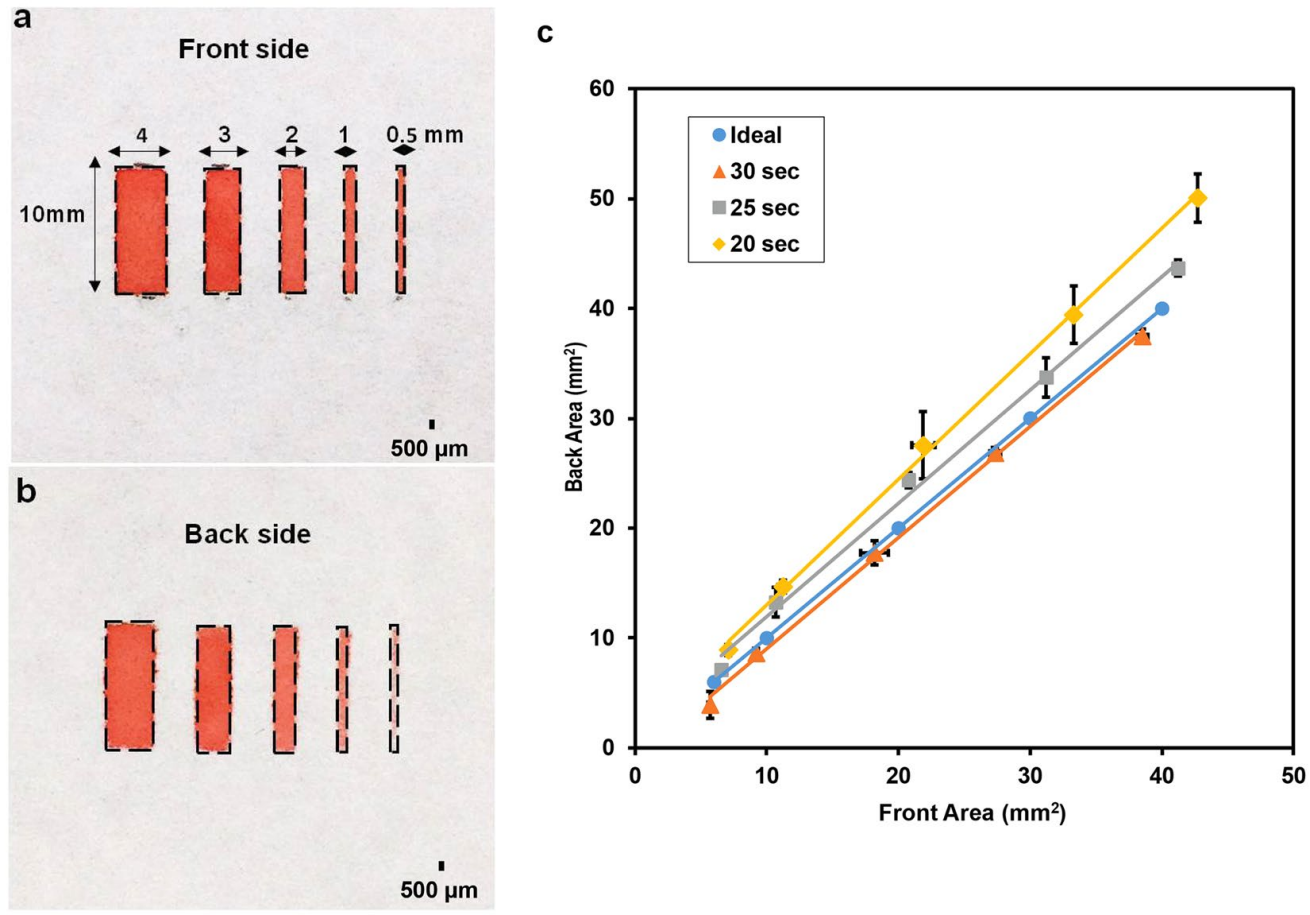
Characterization of C-µPAD technique by controlling CVD duration and channel area (**a**) Front side of the patterned chromatography paper at 30 seconds of CVD duration. (**b**) Back side of the patterned chromatography paper at 30 seconds of CVD duration. (**c**) Different dimensions of channels (4, 3, 2, 1, 0.5 mm width × 10 mm length) were analyzed with different CVD duration (20, 25, and 30 seconds).

Optimal CVD duration with various sized rectangle patterns was characterized to determine resolution of this C-µPAD fabrication technique. Figure 2 shows the result of this experimental characterization. Figure 2(a) and (b) present the front and back side of the patterned papers. Both sides formed the proper hydrophobic barriers without any spreading issues. Figure 2(c) shows the relationship between channel area and CVD duration for the respective channel size. As shown in Fig. 2(c), 30 seconds CVD duration at 53 °C is the best fit with the ideal case which is the same hydrophilic area for both front and back side of the patterned paper. For CVD durations longer than 30 seconds, the hydrophilic area decreased because the hydrophobic reagent penetrated the hydrophilic barrier under the vinyl tape patterns. For CVD durations shorter than 30 seconds, the spreading degrees on both sides were larger than the original area since the vaporized TCS molecules could not penetrate through the paper completely to form hydrophobicity on the back side of the paper. From this characterization, with 30 seconds CVD duration at 53 °C, the C-µPAD enabled us to fabricate as low as 500 μm width channel with the best hydrophilic and hydrophobic contrast. This fabrication limit is due to aspect ratio of channel width and paper thickness under penetration speed of vaporized TCS molecules. Using current chromatography paper, we achieved aspect ratio up to 0.4 on the 200 μm thick chromatography paper. Random mobility of TCS vapor molecules in vacuum chamber results in relatively low aspect ratio. The aspect ratio of this fabrication method can be improved by choosing a thinner paper having high density of hydroxyl functional groups.

**Figure 3:**
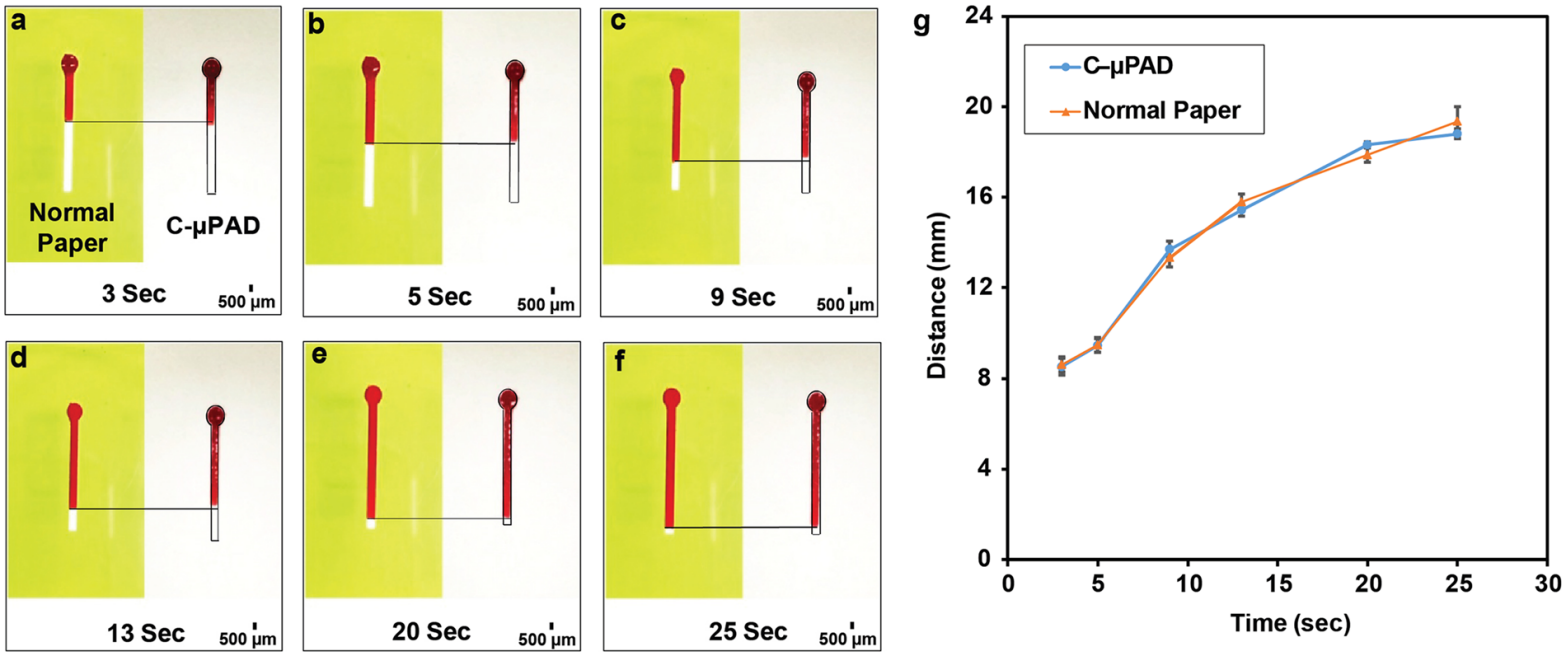
Distance vs. time analysis on normal chromatography paper and patterned fluidic device with red dye solution. (**a**–**f**) Red dye color solution was applied onto both untreated chromatography paper and treated paper-based microfluidic device to demonstrate the different time point flow rate. (**g**) The distance that red dye color travels inside the channel of C-µPAD in terms of time was then compared with the data of fluid flows in a normal chromatography paper.

The flow velocity of C-µPAD was compared with that of a normal chromatography paper. During the CVD process, vaporized TCS molecules can penetrate from the top or side of masking film. This unwanted exposure influences the wicking properties of the paper which alters fluid transport properties of the C-µPAD. Figure 3(a–d) shows the difference between fluid velocities in a normal chromatography paper and the C-µPAD. There was only ~5% variation for overall velocities as shown in Fig. 3(e). This much variation can originate from paper itself since the internal structure of chromatography paper consists of highly heterogeneous fiber directions, pore size, and pore distribution.

### Assay Demonstrations

#### Glucose Assay

**Figure 4:**
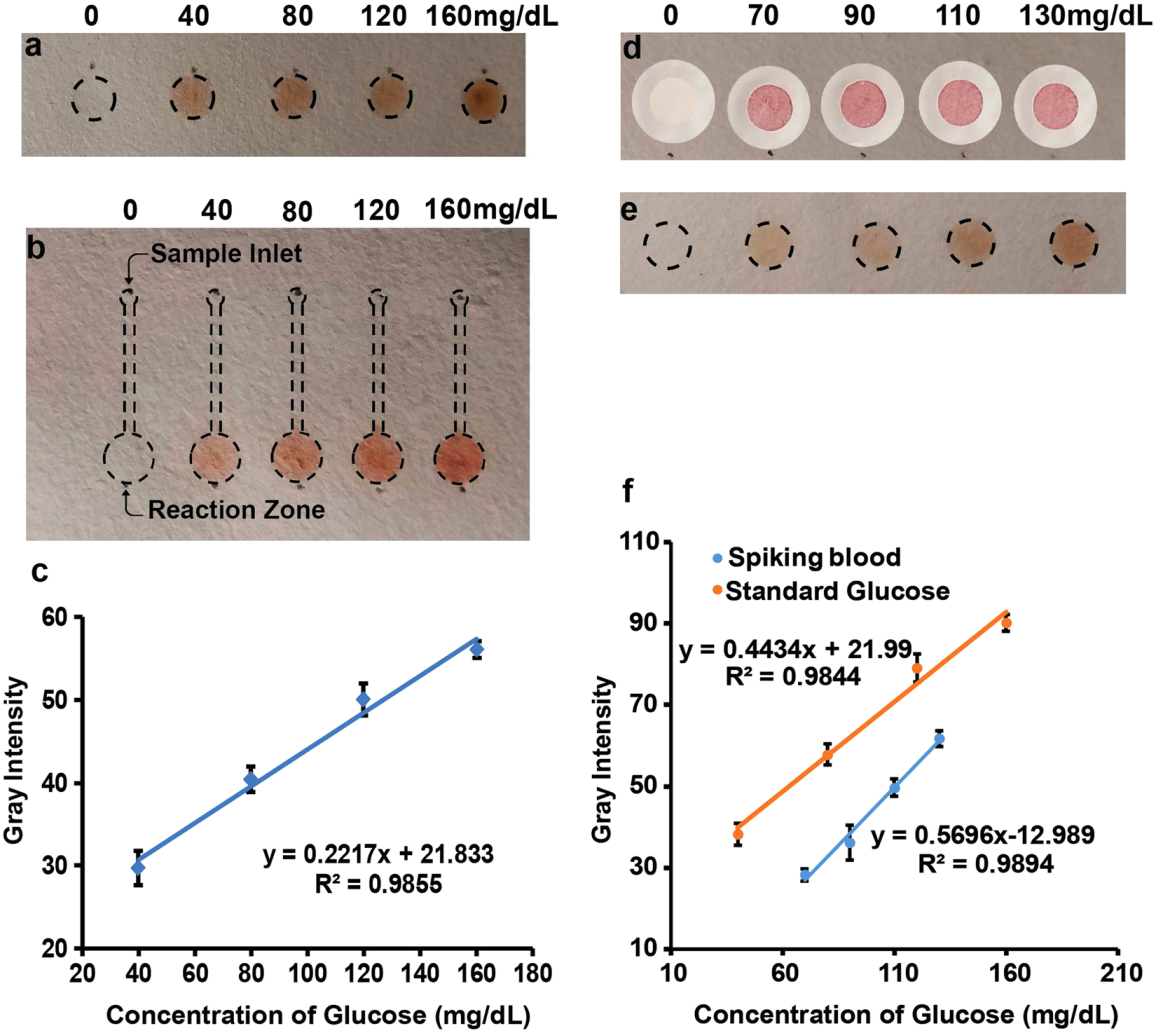
Demonstration of glucose assay on well-spot C-µPAD and lateral flow C-µPAD. (**a**) Different concentrations of glucose solutions from 0 to 160 mg/dL were applied onto each spot on a well-spot C-µPAD. (**b**) Glucose assays with various concentrations of glucose from 0 to 160 mg/dL were analyzed on lateral flow C-µPAD. Assay reagents were added into the reaction zone, the other end of channel allowed to flow glucose solution freely into the reaction zone. (**c**) This plot shows a linear relationship between various concentrations of glucose and their differential gray intensity in lateral flow C-µPAD. (**d**) Front side of C-µPAD after extracting plasma separation from the human blood. Each spot shows similar color since each glucose-spiked sample uses the same amount of blood. (**e**) Well-spot glucose assays from various concentrations of glucose-spiked blood samples. (**f**) Glucose assay results on the well-spot C-µPAD using standard glucose samples and glucose-spiked whole blood samples. These results show a strong linear relationship between glucose concentrations and gray intensity.

To show bioassay capabilities of C-µPAD, glucose assays were demonstrated on well-spot C-µPAD and lateral flow C-µPAD platforms using standard glucose samples. Figure 4(a) shows that color intensity increases as glucose concentration increases from 0 to 160 mg/dL in glucose well-spot C-µPAD. Based on the LOD calculation^33^, 13 mg/dL of LOD was achieved in well-spot C-µPAD, which is equivalent to the LOD of the commercially available glucose meter (AccuCheck). Furthermore, a lateral flow glucose assay was developed by using a dumbbell-shaped channel. In the reaction zone of the lateral flow C-µPAD, 5 µL of assay reagent was immobilized by spotting 4 times since the reaction zone can accommodate only 1.25 µL. For a glucose assay using the lateral flow C-µPAD, 2.5 µL of glucose sample was then applied on the sample inlet, allowed to flow, and reacted in the detection zone. Figure 4(b) shows the gradient of color intensity depending on the glucose concentration. Figure 4(c) clearly indicates the differences between the color intensity for each concentration of glucose in lateral flow C-µPAD. The lateral flow assay was successfully demonstrated with the same range of glucose concentration with well-spot C-µPAD glucose assay and 23 mg/dL of LOD was achieved. To determine the correlation between 96-well plate and lateral flow C-µPAD methods, the results from both measurements were compared. As shown in Supplementary Figure S2, lateral flow C-µPAD data has a good agreement with the standard 96-well plate assay measured by spectrophotometer. Even though the same concentration of glucose was used for both well-spot C-µPAD and lateral flow C-µPAD, the well-spot C-µPAD showed higher color intensity. For well-spot C-µPAD, there was no volume loss since all applied glucose samples reacted with immobilized assay reagents. For lateral flow C-µPAD, 2.5 µL of glucose sample was applied on the sample inlet, which had to flow through the channel to reach the reaction zone. From volume calculation, ~0.8 µL of glucose samples reacted with the immobilized assay reagents.

To demonstrate the C-µPAD ability for point-of-care diagnostics (POC), a human blood glucose assay on well-spot C-µPAD was conducted. A plasma separation membrane (Pall Corporation) was fixed on the front side of the well-spot C-µPAD to extract plasma from the blood shown in Fig. 4(d). Figure 4(e) shows the assay results on the back side of the well-spot C-µPAD from glucose-spiked blood samples. As expected, overall color intensity increases as the total amount of glucose increases from glucose-spiked blood samples. Compared to the standard well-spot glucose assay in Fig. 4(f), the color intensities from the blood samples are less than those of the standard glucose samples due to differences in fluidic properties and mild reaction inhibitors in the extracted plasma^34^. Even overall intensity is shifted down a bit, sensitivity from the blood samples is almost identical with that from the standard glucose samples.

#### Immunoassay

**Figure 5:**
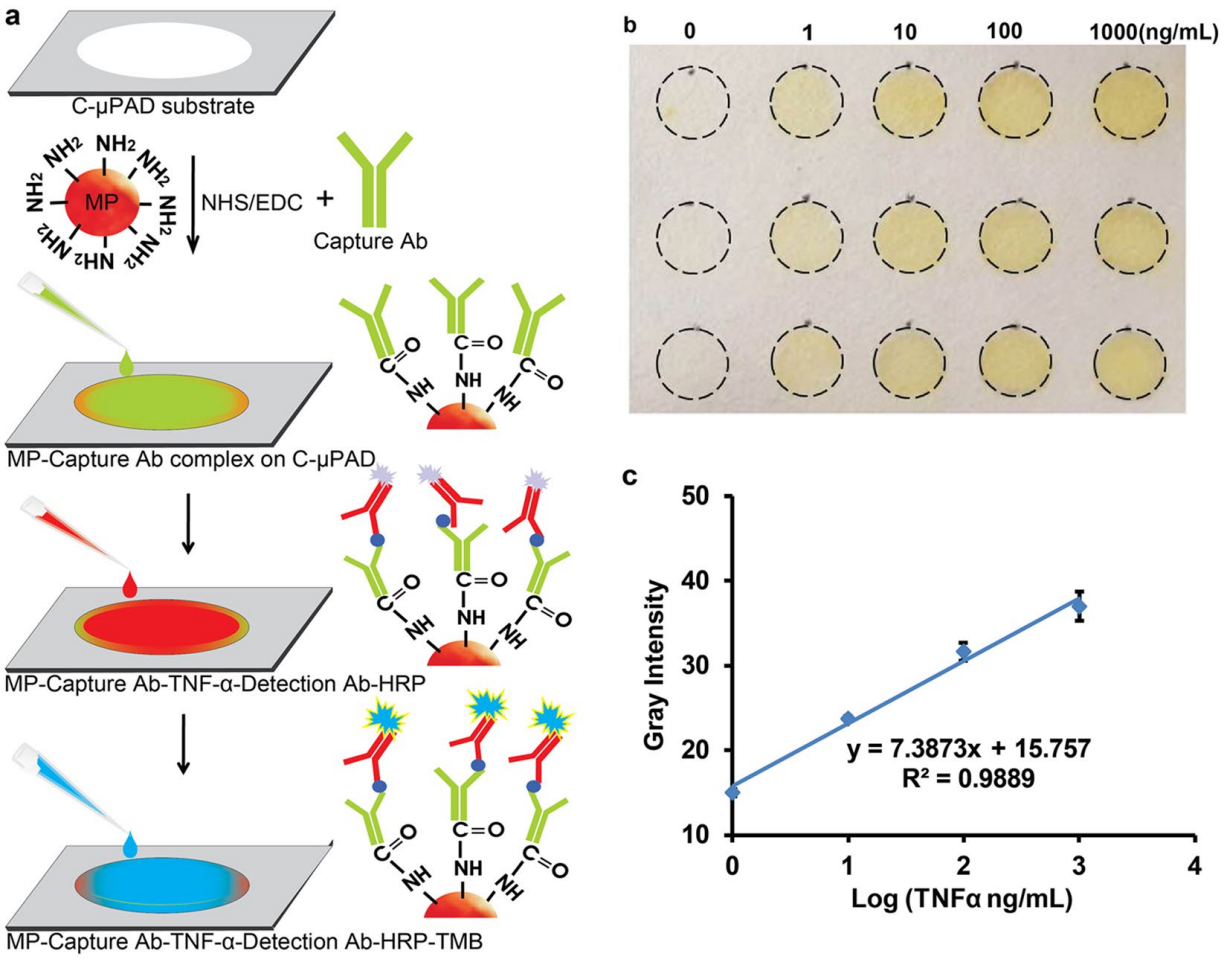
(**a**) Procedure of immunoassay on C-µPAD. (**b**) C-µPAD shows a clear concentration gradient after the assay with different concentration of TNFα (0 ng/mL~1000 ng/mL). (**c**) Immunoassay results acquired by a smartphone camera and analyzed by image J software. These results show log-linear relationship between TNFα concentrations and differential gray value.

In addition to glucose assays, a sandwich immunoassay for TNFα quantification was demonstrated on well-spot C-µPAD by following the procedure shown in Fig. 5(a). 1 µm sized amine terminated magnetic particles was suitable to apply on the well-spot C-µPAD since the pore size of the paper is in the range of ~1 µm and can physically trap the beads. EDC/NHS was used to activate the carboxyl group of human anti-TNFα capture antibody that would lead to covalent conjugation between human anti-TNFα antibody and amine-terminated magnetic particles. The immune complex was formed on the amine functionalized magnetic particles. On positive well-spot, the appearance of blue color confirmed the formation of immune complex. Further, TMB stop solution interrupted the reaction which would lead color changes from blue to yellow as shown in Fig. 5(b). The intensity of yellow color developed in each well-spot was proportional to the amount of TNFα formed on the well-spot C-µPAD. Figure 5(c) shows a log-linear correlation for various concentrations of TNFα demonstrating that the color intensity increased to the concentration ranges of 1~1000 ng/mL. From this TNFα immunoassay demonstration, 3 ng/mL of LOD was achieved under well-spot C-µPAD. This result of immunoassay in well-spot C-µPAD is less sensitive than conventional ELISA. However, in conventional ELISA techniques, larger volume of analytes and reagents including long incubation due to the diffusion limit are required. Compared to the traditional ELISA, C-µPAD decreased the reaction time significantly since this C-µPAD offered 3-dimensional matrix to create immune complex by transporting all liquid samples with wicking force. Furthermore, less than 15 µL of total reagents was sufficient to carry out this sandwich immunoassay on well-spot C-µPAD. Based on this concept, we can implement the C-µPAD for the detection of cardiovascular disease markers, respiratory disease markers, HIV, HBV, etc., using blood or saliva with POC diagnostic approach. As well, the requirements of small reagent volume, rapid analysis, portable, multiplex detection and low cost are the important qualities to POC diagnostics in both developed and developing countries.

#### Heavy metal detection

**Figure 6:**
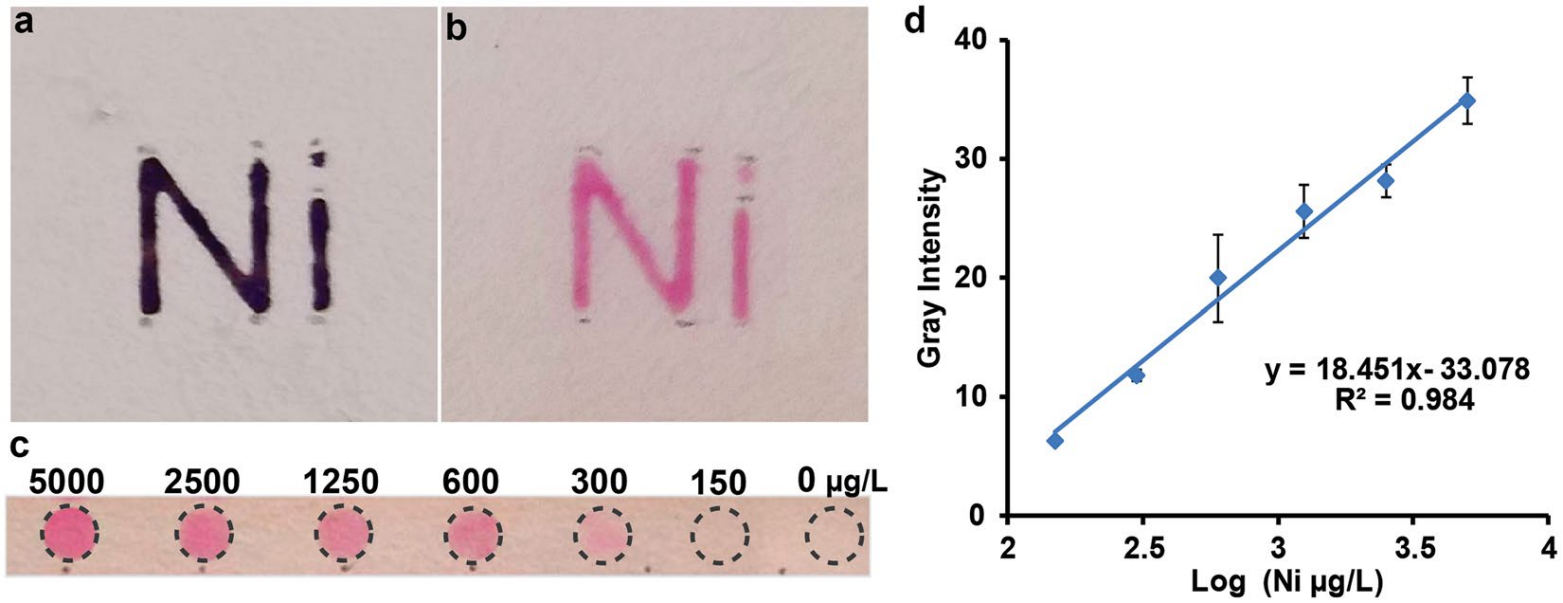
(**a**) Immobilization of amine functional group on “Ni” symbol patterned C-µPAD (**b**) Ni detection on “Ni” symbol patterned C-µPAD (**c**) Various concentrations of Ni on the well-spots C-µPAD (**d**) A standard curve for Ni detection. Gray intensity increases with a log-linear relationship with concentration of Ni.

Heavy metal detection on C-µPAD was demonstrated on both well-spot and chemical symbol patterned C-µPAD. Nickel (Ni) is one of the heavy metals that could enter into the environmental water through mining, industrial activities, and leaching from wastes. Generally, colorimetric reagents of heavy metal detection spread easily towards the edge of reaction zone on the µPADs which generate cross-reaction and poor detection sensitivity^35^. To resolve this critical problem, we performed amine functionalization on the patterned C-µPAD with APTES by condensation between APTES and OH groups of cellulose fibers on the chromatography paper. Amine functionalization on the “Ni” patterned C-µPAD was then confirmed by a standard ninhydrin test. As shown in Fig. 6(a), bluish-purple color was developed only on the letter “Ni” and hydrophobic barrier on the C-µPAD was maintained well even after heating at 95 °C. Generally, the most common fabrication for µPAD is to use a wax printing technique which is thermally unstable above 60 °C^22^. For the common µPAD, selective silane functionalization will be extremely challenging due to thermal instability of printing material. Using this unique property of C-µPAD, Ni detection was demonstrated on amine functionalized well-spot C-µPAD by coupling dimethylglyoxime (DMG). DMG is a colorimetric reagent which can be covalently attached on the amine functionalized C-µPAD and reacted with nickel to form DMG-Ni complex presented in Fig. 6(b). By adding various concentration of Ni solution, color intensity is proportional to concentration of Ni as shown in Fig. 6(c). A standard curve of Ni detection is presented in Fig. 6(d). From this Ni detection demonstration, we were able to detect as low as 150 μg/L of Ni concentration which shows two folds improvement with the previous report^36, 37^. This heavy metal assay demonstration proves that the C-µPAD enables us to use for silane functionalization which requires thermal condensation to form covalent bonds. In addition, hydrophobicity on the C-µPAD was extremely stable since the TCS molecules altered wetting property of cellulose fibers in the chromatography paper. To estimate overall shelf-life of the C-µPAD, the patterned C-µPADs were stored at ambient conditions for more than six months. There is no significant alteration on hydrophobic barriers for maintaining fluidic patterns on the patterned C-µPADs.

## Conclusion

Here, we demonstrated a CVD method to form hydrophilic-hydrophobic barriers by depositing vaporized TCS on a chromatography paper. TCS treated region on the paper established strong hydrophobic layers compared to an untreated chromatography paper. This C-µPAD technique is a simple, rapid, and thermally insensitive procedure compared to the previously reported µPAD fabrication techniques. In addition, this C-µPAD technique has a high commercial potential with low-cost fabrication and mass production capabilities. With this C-µPAD technique, various bioassays such as glucose and immunoassay have been demonstrated with clinically relevent LODs. Furthermore, all assays were performed with ~5 µL target sample volume without the use of a spectrophotometer or other advanced equipment. All assay demonstrations show that this C-µPAD enables us to revolutionize POC diagnostics by further validations with clinical samples. Additional assay demonstrations such as cardiac panel, cytokines, and liver panel screenings are required to show adaptability of this platform. The heavy metal detection analysis proves that the C-µPAD is capable to detect the environmental contaminants by immobilizing amine functional group using thermal condensation. Functionalizing the C-µPAD using thermal condensation is a significant achievement to improve stability, sensitivity, and specificity for point-of-care chemical and biological analysis. With extension, other functional groups such as carboxyl and thiol terminated silane compounds can be easily immobilized for conjugating nucleic acids, proteins, hormones, drugs, and enzymes selectively for multiplexing biological and chemical analysis.

## Methods

### Materials and Equipment

Trichlorosilane (97%) was purchased from Sigma-Aldrich and used as hydrophobic agent. D-(+)-Glucose (99.5%), glucose oxidase/peroxidase (from Aspergillus niger, 215 U mg^−^1), and o-dianisidine were purchased from Sigma-Aldrich to carry out the enzymatic glucose assay. Human whole blood with potassium oxalate/sodium fluoride anticoagulant was purchased from BioreclamationIVT and Vivid Plasma Separation GX membrane was obtained from Pall Corporation. Human tumor necrosis factor alpha (TNFα) protein, anti-human TNFα antibody, and biotinylated anti-human TNFα antibody were purchased from Abcam for immunoassay demonstration. Streptavidin conjugated horseradish peroxidase (HRP), Tetramethylbenzidine (TMB), stop solution (contains 0.16M sulfuric acid), and sample diluents were purchased from Thermofisher Scientific. Food coloring dye (McCormick & Company, Inc., MD) and Whatman No. 1 chromatography paper (Carolina biological, NC) were used for characterization and fabrication of C-µPAD.

### C-µPAD Fabrication and Characterization

#### Fabrication of C-µPAD

The procedure of C-µPAD fabrication is schematically represented in Fig. 1(a). At first, a desired fluidic pattern was drawn in AutoCAD and cut on a vinyl tape by using a vinyl cutter (Roland RX-1). This patterned tape was transferred to 4.5 × 5 cm chromatography paper which was then fixed on top of a vacuum chamber containing 100 µL of TCS solution on a 53 °C heat block. In this CVD process, pre-vacuum was applied for 90 seconds and maintained to vaporize liquid TCS before actual deposition was performed to obtain ideal hydrophobic barriers. After CVD process, the patterned paper was heated on a hot plate for 1 minute at 75 °C to remove the vinyl tape. The paper area under the tape would be hydrophilic while all other areas of the paper would be hydrophobic. This chemically patterned chromatography paper was further evaluated by using color dyes shown in Fig. 1(b–d).

#### Characterization of C-µPAD

CVD duration was characterized by measuring contact angle on hydrophobic area of the paper and the patterned channel size of C-µPAD. To characterize hydrophobicity of the exposed paper with respect to CVD duration, a quarter of original chromatography paper (11 cm × 11 cm) was fabricated by CVD method for times varying from 10 seconds to 60 minutes. After CVD treatment, each treated paper was cut and fixed onto a slide glass for the contact angle measurement using a goniometer (Wet Scientific). 5 μL of DI water was dropped on the surface of each treated paper to measure the contact angle.

In addition, the relationship between CVD duration and fabrication resolution of the C-µPAD was investigated. At first, rectangular channel patterns with dimensions of 4, 3, 2, 1, and 0.5 mm in width with 10 mm in length were prepared with the vinyl cutter and performed CVD process with CVD duration of 20, 25, and 30 seconds. After processing this fabrication, food dye solution was applied on each channel to determine total spreading area on front and back side of the channel in each CVD duration. Furthermore, flow velocity within the hydrophilic channel was characterized and compared with an intact chromatography paper. A C-µPAD was created with a 20 mm dumbbell-shaped channel and a similar shape was cut on a chromatography paper using the vinyl cutter. Food coloring dye solution was applied onto both C-µPAD and normal chromatography paper at the same time, and a videotape was recorded in order to compare the flow velocity with each other.

### Glucose Assay

#### Glucose assay on well-spot C-µPAD

D-(+)-Glucose powder was dissolved in deionized (DI) water and serially diluted to prepare 0~160 mg/dL concentration of a standard glucose solution. Then, the combination of glucose oxidase/peroxidase and o-dianisidine was prepared to serve as the assay reagent. 5 μL of assay reagent was physically immobilized and dried onto each well of well-spot C-µPAD. 1 μL of standard glucose solutions with various concentrations was then applied on each well-spot. After 10 minutes, oxidized o-dianisidine produced brown color on each well depending on the glucose concentration. Optical images of this well-spot glucose assay were converted to the 8-bit gray scale and gray intensity of each well was measured by imageJ. All well-spot data was adjusted with background (0 mg/dL) to obtain the absolute values.

#### Glucose assay on lateral flow C-µPAD

A lateral flow glucose assay using lateral flow C-µPAD was developed to demonstrate the additional step of sample transport to the spotted detection reagents. A lateral flow C-µPAD with dumbbell-shaped patterns was created for this lateral flow glucose assay. 5 μL of assay reagent was physically immobilized and dried onto reaction wells. 2.5 μL of various concentration of glucose solutions such as 0, 40, 80, 120, and 160 mg/dL was then dropped onto the sample inlet and allowed to flow freely to each reaction well. After 10 minutes, oxidized o-dianisidine produced brown color at reaction well depending on the glucose concentration. The aforementioned process was followed to measure the gray intensity. Furthermore, the standard glucose assay was performed in a 96-well plate for comparison with the glucose assay on C-µPAD. For this standard assay, the same concentrations with the lateral flow C-µPAD were tested by following a commercial protocol (Sigma Aldrich glucose assay kit) and analyzed by microplate reader (Genesys, Tecan).

#### Whole blood glucose assay on well-spot C-µPAD

D-(+)-Glucose powder was dissolved in phosphate-buffered saline (PBS) and serially diluted for various concentration of 0 mg/dL, 40 mg/dL, 80 mg/dL, and 120 mg/dL and then spiked into 140 mg/dL of human whole blood with the volume ratio of 1:1. 5 μL of assay reagent was physically immobilized on each well of well-spot C-µPAD and allowed to dry in the room temperature. 2.5 mm in diameter of Vivid Plasma Separation GX membrane was soaked with PBS and dried in the room temperature before assembly. The dried plasma separation membrane was then attached on each well spot with a vinyl ring-shaped tape. 3 μL of each spiked blood sample was applied on each membrane to quantify glucose level. After 5 minutes, brown color spot was observed on back side on the each well and gray intensity of each well was analyzed as the aforementioned procedure.

### Immunoassay on Well-spot C-µPAD

Well-spot C-µPAD was developed with 4 mm in diameter well-spot array pattern. For the immunoassay, 1 µg/mL TNFα antibody was incubated with 1 µg/mL of EDC/NHS for 30 minutes and applied on the 1 µm size of amine functionalized magnetic particles. The magnetic particles were physically immobilized on the well-spot C-µPAD. 1 µL of various concentrations including 1, 10, 100, and 1000 ng/mL of human TNFα was then applied on each well-spot and incubated for 10 minutes. Then, 1 µg/mL of human TNFα biotinylated antibody, and followed by 2 µL of streptavidin- HRP reagents was applied on the substrate; again, the substrate was incubated for 10 minutes at room temperature. Subsequently, the well-spot C-µPAD was placed into a petri dish, covered in 500 µL of PBS, and rinsed 2 times to remove unbound proteins. Further, the spots were dried using laboratory wipes. 2 µL of TMB substrate was then applied on each spot and incubated for 10 minutes to induce the peroxidase reaction. 2 µL of TMB stop solution was then added in each spot to stop the reaction that produced yellow color based on the antigen concentration. For a negative control, 1% of BSA solution was immobilized on the amine-terminated magnetic particles instead of TNFα and their respective antibodies. Finally, TMB solution was applied on the control spot. Furthermore, gray intensity of each well was analyzed using ImageJ after acquiring high resolution photographs and converting to gray scale.

### Heavy Metal Detection

#### Immobilization of the amine functional group by thermal condensation

Chemical symbol “Ni” and well-spot patterned C-µPADs were created using CVD process shown in Fig. 1. 1.5 µL of 3% (3-aminopropyl) tri-ethoxysilane (APTES) was then applied on the patterned chemical symbol and well-spot area and heated at 95 °C for condensation. These C-µPADs were then washed with DI water to remove the unbound APTES and gently dried on a hot plate at 50 °C. After these functionalization steps, amine immobilization was confirmed by a standard ninhydrin colorimetric test.

#### Nickel detection on thermally stable C-µPAD

On amine-modified chemical symbol “Ni” and 4 mm diameter well-spot C-µPAD, 2 µL of colorimetric reagent (10 mg/mL of Dimethyl glyoxime (DMG) in 50% Ethanol) was applied on the amine functionalized area of the C-µPAD. These C-µPADs were then heated at 65 °C for dehydration. On the “Ni” patterned C-µPAD, 5000 μg/L of NiSO_4_ was applied to confirm the colorimetric reaction. On spot-well patterned C-µPAD, various concentrations of NiSO_4_ (5000 μg/L, 2500 μg/L, 1250 μg/L, 600 μg/L, 300 μg/L, 150 μg/L, 0 μg/L (DI water)) were applied on each well on the C-µPAD to obtain a standard curve. All color intensity measurement and analysis methods are the same with previous assays.

## Electronic supplementary material

Supplementary Information
