# Supplementary material for: A Chemically Patterned Microfluidic Paper-based Analytical Device (C-µPAD) for Point-of-Care Diagnostics
Source: Sci Rep. 2017 Apr 26;7:1188. doi: 10.1038/s41598-017-01343-w (PMC5430703; doi:10.1038/s41598-017-01343-w)
Supplement: Supplementary file 1 — Supplementary Information [file 41598_2017_1343_MOESM1_ESM.pdf]

Supplemental information

A Chemically Patterned Microfluidic Paper-based Analytical Device (C-µPAD) for Point-of-Care Diagnostics

Trinh Lam[1#], Jasmine P. Devadhasan[2#], Ryan Howse[2] and Jungkyu Kim[2*]

[1] Department of Chemical Engineering, Texas Tech University, Lubbock, TX

[2]Department of Mechanical Engineering, Texas Tech University, Lubbock, TX

* Address correspondence to: Jungkyu (Jay) Kim

Department of Mechanical Engineering

Texas Tech University

Lubbock, Texas 79409, USA

Phone: (806) 834-6106

E-mail: jungkyu.kim@ttu.edu

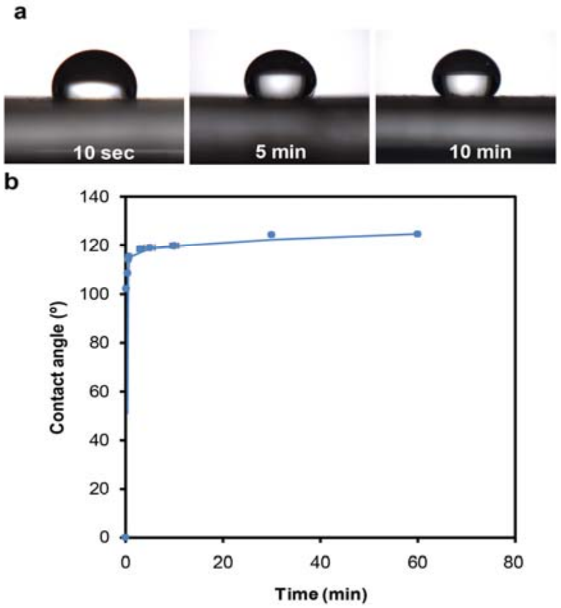

c

Time (min)	Contact Angle	STDEV
0	0	0.0
0.2	102.4	0.3
0.3	108.7	0.3
0.5	114.3	0.1
0.7	115.6	0.5
3.0	118.6	0.6
5.0	119.2	1.1
10.0	119.9	0.9
30.0	124.5	0.0
60.0	124.8	0.4

Figure S1. (a) Water droplet on the surface of treated chromatography paper which was processed with CVD method with different durations. (b) The relationship of contact angle of water droplet and CVD durations (c) Contact angle and their respective standard deviation in terms of CVD duration.

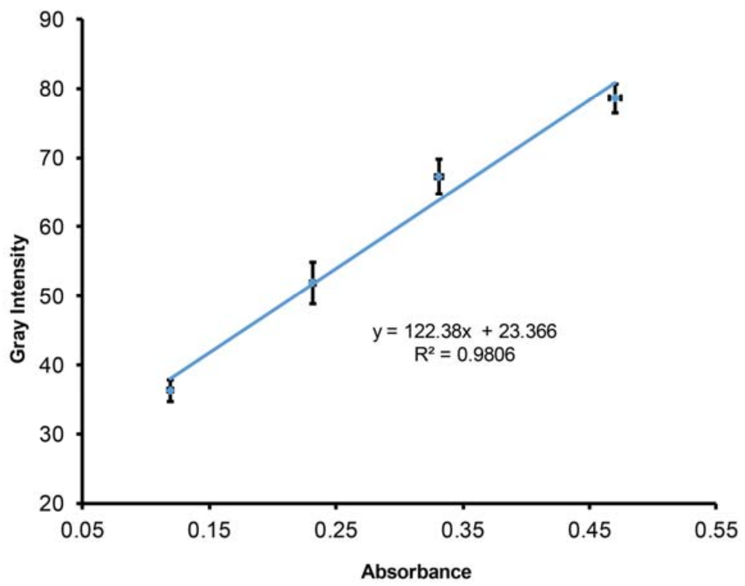

Figure S2. Plot of absorbance obtained from 96-well plate and differential gray intensity value from lateral flow assay for glucose detection. Two results show a linear relationship.
